# Single Chiral Skyrmions in Ultrathin Magnetic Films

**DOI:** 10.3390/ma11112238

**Published:** 2018-11-11

**Authors:** Arantxa R. Aranda, Konstantin Y. Guslienko

**Affiliations:** 1 Dpto. Física de Materiales, Universidad del País Vasco, UPV/EHU, 20018 San Sebastián, Spain; rodrguez.arnzazu@gmail.com; 2 IKERBASQUE, the Basque Foundation for Science, 48013 Bilbao, Spain

**Keywords:** magnetic skyrmion, ultrathin film, micromagnetism

## Abstract

The stability and sizes of chiral skyrmions in ultrathin magnetic films are calculated accounting for the isotropic exchange, Dzyaloshinskii–Moriya exchange interaction (DMI), and out-of-plane magnetic anisotropy within micromagnetic approach. Bloch skyrmions in ultrathin magnetic films with B20 cubic crystal structure (MnSi, FeGe) and Neel skyrmions in ultrathin films and multilayers Co/X (X = Ir, Pd, Pt) are considered. The generalized DeBonte ansatz is used to describe the inhomogeneous skyrmion magnetization. The single skyrmion metastability/instability area, skyrmion radius, and skyrmion width are found analytically as a function of DMI strength d. It is shown that the single chiral skyrmions are metastable in infinite magnetic films below a critical value of DMI $d_{c}$, and do not exist at $d>d_{c}$. The calculated skyrmion radius increases as d increases and diverges at $d\to d_{c}-0$, whereas the skyrmion width increases monotonically as d increases up to $d_{c}$ without any singularities. The calculated skyrmion width is essentially smaller than the one calculated within the generalized domain wall model.

## 1. Introduction

The individual (single) magnetic skyrmions have attracted considerable attention from researchers assuming potential applications in spintronic and information processing devices [1]. To achieve efficient manipulation of the skyrmion spin textures and to realize skyrmion-based low energy consumption devices, it is essential to understand the magnetic skyrmion stability and dynamics, for instance, in ultrathin ferromagnetic films.

The chiral magnetic skyrmions are a kind of magnetic topological soliton [2] in 2D spin systems characterized by a non-zero skyrmion number (topological charge, degree of mapping) defined as $N=\int d^{2}\rho m\cdot \left(\partial_{x}m\times \partial_{y}m\right)/4\pi$, where $m\left(\rho \right)=M\left(\rho \right)/M_{s}$ is the unit magnetization vector, $M_{s}$ is the material saturation magnetization, and ρ=(x,y) are in-plane spatial coordinates. The number N=±1, ±2,… is an integer for an infinite film. This topological charge can be interpreted as a quantized flux of the emergent magnetic field [3] through the film surface, $\Phi =\left|N\right|\Phi_{0}$, where $\Phi_{0}=h/e$ is the flux quantum.

The relativistic Dzyaloshinskii–Moriya exchange interaction (DMI) leads to the stabilization of chiral Neel or Bloch skyrmions with a given sense of the magnetization rotation within their internal configuration [1]. The role of the DMI in skyrmion stabilization was discussed in Refs. [4,5,6,7]. Following the ideas of Dzyaloshinskii [4], in Ref. [5] it was found that adding the term D[m⋅(∇×m)] (linear in spatial derivatives of magnetization) to the magnetic energy density of an infinite cubic ferromagnet leads to the stabilization of an inhomogeneous magnetization texture for any finite value of the DMI parameter *D*. Such terms are allowed in magnetic crystals whose symmetry group lacks the space inversion symmetry operation (e.g., in the B20 cubic crystals MnSi, FeGe, etc. [1]). Then, it was shown [6] that accounting for DMI in the form of the Lifshitz invariants in a bulk uniaxial ferromagnet results in the instability of the uniform ferromagnetic state at $D>D_{c}=\left(4/\pi \right)\sqrt{AK}$, where *A* is the exchange stiffness and *K* is a uniaxial anisotropy constant. The 1D spin spiral becomes the ground state at $D>D_{c}$. Therefore, DMI can stabilize 2D vortices (Bloch skyrmions, in modern terminology) for moderate values of *D*. Ivanov et al. [7] showed that the Bloch skyrmions in infinite films with easy axis anisotropy can be stabilized either by DMI or a high-order exchange interaction. Another kind of single chiral skyrmion (Neel skyrmions) was recently observed at room temperature by Boulle et al. in Pt/Co/MgO [8], Moreau-Luchaire et al. in Ir/Co/Pt [9], Woo et al. in Pt/Co/Ta, Pt/CoFeB/MgO [10], and Pollard et al. in Pd/Co [11] ultrathin multilayer films and dots. These Neel skyrmions are stabilized by the DMI existing at the ferromagnetic metal and heavy metal interface. Such interfacial DMI can be represented as the energy density $\varepsilon_{DMI}=D\left[m_{z}\left(\nabla \cdot m\right)-\left(m\cdot \nabla \right)m_{z}\right]$, where the unit vector **z** is normal to the interface. The DMI lowers the skyrmion energy for the proper skyrmion chirality.

In the case of an infinite ferromagnetic film, the critical *D* value presumably remains the same as for bulk crystals, $D_{c}=\left(4/\pi \right)\sqrt{AK}$, although the effective anisotropy constant *K* is different. The isolated skyrmions are metastable at $D<D_{c}$ at zero external magnetic field, and other configurations (e.g., spin spirals, skyrmion lattices, stripe domains) are stabilized at $D>D_{c}$ [12,13].

In this article, we calculate the magnetic energy of a single chiral skyrmion in ultrathin magnetic film and determine the area of the skyrmion metastability, skyrmion magnetization profiles, and the equilibrium skyrmion radius and width. The case of an effective out-of-plane magnetic anisotropy is analyzed.

## 2. Methods

Let us consider an infinite magnetic film with thickness *L* of about 1 nm, and parameterize the unit magnetization vector by the spherical angles, m=m(Θ,Φ). The spatial distribution of magnetization is assumed to be independent of the thickness coordinate *z*. The angles Θ, Φ are functions of the polar radius vector ρ=(ρ,ϕ) located in the film plane. For this kind of magnetization configuration, the total magnetic energy functional is $E\left[m\right]=L\int d^{2}\rho \varepsilon \left(m\right)$ [6,7], with the energy density

(1) $$\varepsilon \left(m\right)=A{\left(\nabla m\right)}^{2}+\varepsilon_{DMI}(m)-K_{u}m_{z}^{2}+\varepsilon_{m}\left(m\right),$$

where *A* is the material exchange stiffness, $\varepsilon_{DMI}$ is the DMI energy density, with *D* being the DMI parameter, $K_{u}>0$ is the out-of-plane uniaxial anisotropy constant, $m_{z}$ is the magnetization *z*-component, and $\varepsilon_{m}$ is the magnetostatic energy. The interface DMI density is $\varepsilon_{DMI}\left(m\right)=D\left[m_{z}\left(\nabla \cdot m\right)-\left(m\cdot \nabla \right)m_{z}\right]$ for the Neel skyrmions, or $\varepsilon_{DMI}\left(m\right)=D\left[m\cdot \nabla \times m\right]$ for the Bloch skyrmions, in thin films of the B20 cubic crystals.

The magnetostatic energy $\varepsilon_{m}\left(m\right)$ is non-local in a general case. The volume and surface magnetic charges contribute to the magnetostatic energy. However, within the limit of ultrathin film, the volume magnetic charges can be neglected, and only surface magnetic charges on the film top/bottom surfaces related to the out-of-plane magnetization component $m_{z}$ contribute to the magnetostatic energy. Then, the magnetostatic energy density can be essentially simplified and written in the local form $\varepsilon_{m}\left(m\right)=\mu_{0}M_{s}^{2}m_{z}^{2}/2$ [2,7] for both kinds of skyrmion. Therefore, the energy is accounted via an effective uniaxial anisotropy constant $K=K_{u}-\mu_{0}M_{s}^{2}/2>0$. We also define the characteristic magnetic material length $l=\sqrt{A/K}$, and the reduced dimensionless DMI strength d=Dl/A.

We search for axially symmetric inhomogeneous magnetization configurations (**m** depends only on the radial coordinate ρ), that is, the magnetization angles are Θ=Θ(ρ), $\Phi =\varphi +\varphi_{0}$ ($\varphi_{0}=0,\;\pi$ for the Neel skyrmions or $\varphi_{0}=\pm \pi /2$ for the Bloch skyrmions). The total skyrmion magnetic energy as a functional of the skyrmion magnetization is represented by the polar magnetization angle Θ(ρ), E=E[Θ(ρ)]. The DMI energy depends on the skyrmion chirality ℂ=±1, which is defined as $\mathbb{C}=\sin\varphi_{0}$ for the Bloch skyrmions and $\mathbb{C}=\cos\varphi_{0}$ for the Neel skyrmions. The sign of DMI strength D depends on the particular ferromagnetic material. Appropriate choice of the sign of chirality at given D ensures that the product Dℂ corresponds to negative DMI energy. We use the total reduced energy of the radially symmetric Bloch or Neel skyrmion (in units of 2πAL)

(2) $$E\left[\Theta \left(r\right)\right]=\int_{0}^{\infty }drr\left[{\left(\Theta_{r}^{\prime }\right)}^{2}+\left(\frac{1}{r^{2}}+1\right)\sin^{2}\Theta +d\mathbb{C}\left(\Theta_{r}^{\prime }+\frac{1}{r}\sin\Theta \cos\Theta \right)\right],\;r=\frac{\rho }{l},$$

which depends only on one material parameter: the reduced DMI strength, *d*.

The simplest magnetization distribution Θ(ρ)=0 corresponds to the energy E[0]=0 and describes the magnetic film ground state. However, there are metastable magnetization configurations with non-trivial dependence Θ(ρ), which can be found from the solution of the Lagrange–Euler equation corresponding to the energy functional given by Equation (2). The Lagrange–Euler equation is a non-linear differential equation and cannot be solved analytically. Therefore, we use the different approximate solutions below or trial functions for the skyrmion magnetization profile Θ(ρ). Introducing a trial function (skyrmion ansatz) to the energy functional (2), one can calculate the energy of the skyrmion configuration. The simplest trial function, sometimes used in the theory of domain walls and skyrmions [12], is a linear ansatz: $\Theta \left(\rho \right)=\pi \left(1-\rho /2R_{s}\right)$ if $\rho \leq 2R_{s}$ ($R_{s}$ is the skyrmion radius), and Θ(ρ)=0 otherwise. The simplicity of this ansatz allows conduction of the integration in Equation (2) to get the energy $E_{lin}\left(r_{s}\right)=\lambda +r_{s}^{2}-\pi dr_{s}$, where d>0, λ=6.154. The skyrmion equilibrium radius $r_{s}=R_{s}/l$ within the model is $r_{s}=\pi d/2$, and the skyrmion energy is $E_{lin}=\lambda -\pi^{2}d^{2}/4$. The linear model predicts that the skyrmion is in a metastable state at $d<2\sqrt{\lambda }/\pi \approx 1.58$ and that its energy is lower than the energy of the collinear out-of-plane magnetization state at $d>2\sqrt{\lambda }/\pi$.

We can write the Lagrange–Euler equation for the function Θ(ρ) to minimize the skyrmion energy (2) using the substitution tan(Θ(r)/2)=exp(−f(r)) [14]. The boundary conditions for the function Θ(ρ) are Θ(0)=π and Θ(∞)=0 [2,15] or f(0)=−∞ and f(∞)=∞. We define the skyrmion radius by the equation $m_{z}\left(R_{s}\right)=0$, $\Theta \left(R_{s}\right)=\pi /2$ or $f\left(r_{s}\right)=0$, where the reduced radius is $r_{s}=R_{s}/l$.

The approximate solution of the Lagrange–Euler equation at r>>1, far from the skyrmion center r=0, and *d* = 0, is $f\left(r\right)=\left(r-r_{s}\right)$. This is an often-used radial domain wall ansatz taken from the theory of bubble domains in infinite films [16]. This ansatz does not satisfy the boundary condition f(0)=−∞, resulting in singularity of the exchange energy at r=0. Many authors, including Rohart et al. [17] and Buettner et al. [18], considered the skyrmion magnetization configuration as a circular domain wall (DW) located at the skyrmion radius position $R_{s}$, described by the singular domain wall ansatz $\tan\left(\Theta \left(\rho \right)/2\right)=\exp\left(\pm \left(\rho -R_{s}\right)/\Delta \right)$, where Δ is the wall width. In the limit of large radius skyrmion with a sharp edge $R_{s}/\Delta >>1$, the radial DW model becomes asymptotically exact. Recently, it was generalized by Kravchuk et al. [15] considering the domain wall width as a variable δ different from its nominal value $\Delta =\sqrt{A/K}$. The generalized DW ansatz can be used with caution only within the limit $r_{s}/\delta >>1$ (i.e., for the large radius skyrmions) if one conducts integration in Equation (2) in the interval $r\in \left[r_{s}-\delta ,\;r_{s}+\delta \right]$ near the skyrmion edge. To avoid singularity at the origin r=0 and describe the whole range of the skyrmion radii $r_{s}$, we use the trial function $f\left(r\right)=\ln\left(r/r_{s}\right)+\left(r-r_{s}\right)/\delta$ suggested by DeBonte [19] to describe the bubble domains in infinite films. Although such a function is not a solution of the Lagrange–Euler equation, it is evident that f(r) leads to finite exchange energy and satisfies the boundary conditions.

Below, we use the generalized DeBonte ansatz $f\left(r\right)=\ln\left(r/r_{s}\right)+\left(r-r_{s}\right)/\delta$, where the skyrmion radius $r_{s}$ and the skyrmion width δ are variable and depend strongly on the DMI strength, *d*. The equalities cosΘ(r)=tanhf(r), sinΘ(r)=1/coshf(r) allow us to calculate the skyrmion energy (2) and find the areas of the skyrmion metastability/stability. We consider that a skyrmion’s state is stable when it has the lowest energy (ground state) in comparison with other magnetization states. A skyrmion state is metastable when it corresponds to a minimum of the magnetic energy, however, its energy is higher than that of some other magnetization configurations (a local minimum of the energy). The Bloch (Neel) skyrmion energy $E\left(r_{s},\;\delta \right)$ within the generalized DeBonte model is a function of two parameters, $r_{s}$ and δ. Accounting $\Theta_{r}^{\prime }=-\left(1/\delta +1/r\right)\sin\Theta$ we rewrite the skyrmion exchange energy in the form

(3a) $$E_{ex}\left(r_{s},\;\delta \right)=\int_{0}^{\infty }dr\frac{r}{\cosh^{2}f\left(r\right)}\left[{\left(\frac{1}{\delta }+\frac{1}{r}\right)}^{2}+\frac{1}{r^{2}}\right].$$

The exchange energy (3) was calculated by DeBonte, yielding the simple expression

(3b) $$E_{ex}\left(\xi \right)=4+{\left(1-\frac{1}{\xi }\right)}^{2}\ln\left(1+e^{2\xi }\right),$$

where $\xi =r_{s}/\Delta \geq 1$ is the reduced skyrmion radius, and $1/\Delta =1/r_{s}+1/\delta$ is the reduced inverse skyrmion width. In the limit of small radius skyrmion $r_{s}\to 0$, when the exchange energy dominates over other contributions to the energy density, the exchange energy is reduced to the well-known Belavin–Polyakov soliton limit [20], $E_{ex}\left(\xi \to 1\right)=4$, which is determined solely by the skyrmion charge |N| (|N|=1 for the skyrmions considered here).

The magnetic anisotropy and DMI energy can be represented using DeBonte ansatz as

(4) $$E_{an}\left(\xi ,r_{s}\right)=r_{s}^{2}F_{a}\left(x\right),\;E_{DMI}\left(\xi ,r_{s}\right)=-d\mathbb{C}r_{s}F\left(x\right),$$

where *x* = *ξ* − 1, and the functions *F_a_*(*x*), *F*(*x*) are defined as integrals

$$F_{a}\left(x\right)=\int_{0}^{\infty }d\rho \rho \frac{1}{\cosh^{2}f\left(\rho ,x\right)},\;F\left(x\right)=\int_{0}^{\infty }d\rho \frac{1}{\cosh f\left(\rho ,x\right)}\left[1+x\rho -\tanh f\left(\rho ,x\right)\right],\;f\left(\rho ,x\right)=\ln\rho +x\left(\rho -1\right).$$

The function F(x)>0, therefore we chose the sign of dℂ>0 and below use the substitution dℂ→d.

## 3. Results and Discussion

The total skyrmion magnetic energy within the model can be represented as function of two variable parameters, *ξ* and Δ:

(5) $$E\left(\xi ,r_{s}\right)=4+{\left(1-\frac{1}{\xi }\right)}^{2}\ln\left(1+e^{2\xi }\right)+r_{s}^{2}F_{a}\left(\xi -1\right)-dr_{s}F\left(\xi -1\right).$$

The equation $\partial E\left(\xi ,r_{s}\right)/\partial r_{s}=0$ leads to $r_{s}\left(\xi \right)=dF\left(\xi -1\right)/2F_{a}\left(\xi -1\right)$ and allows us to exclude $r_{s}$ from the minimization procedure and write an analytical equation for the equilibrium skyrmion radius as an inverse function of the DMI parameter *d*(*ξ*)

(6) $$d^{2}\left(\xi \right)=\frac{\partial E_{ex}}{\partial \xi }\frac{2F_{a}^{2}\left(x\right)}{F\left(x\right)\left[F^{\prime }\left(x\right)F_{a}\left(x\right)-\frac{1}{2}F\left(x\right){F_{a}}^{\prime }\left(x\right)\right]}.$$

It immediately follows from Equation (6) that the reduced skyrmion radius ξ is a function of $d^{2}$, $\xi =\phi \left(d^{2}\right)$, and for large radius skyrmions ξ>>1, $d\left(\xi >>1\right)\to d_{c}=4/\pi$, or the equilibrium skyrmion reduced radius diverges, ξ(d)→∞, at $d\to d_{c}-0$. In the vicinity of $d_{c}$, Equation (6) yields simple expressions for the equilibrium skyrmion radius $\xi \left(d\right)=1/\sqrt{1-{\left(d/d_{c}\right)}^{2}}$, width $\Delta \left(d\right)=d/d_{c}$, and the energy $E\left(d\right)=4\sqrt{1-{\left(d/d_{c}\right)}^{2}}$. These expressions coincide with ones calculated within the generalized DW model by Kravchuk et al. [15]. At ξ>>1 $F_{a}\left(\xi -1\right)=\xi^{-2}\ln\left(1+\exp\left(2\xi \right)\right)\approx 2/\xi$, $F\left(\xi -1\right)\approx \pi +O\left(e^{-\xi }\right)$, the skyrmion energy is essentially simplified, $E\left(\xi ,r_{s}\right)=2\xi -\pi dr_{s}+2\left(1+r_{s}^{2}\right)/\xi$, and is reduced to one, accounted in the generalized DW model. We note that the DMI and anisotropy energies are proportional to $r_{s}$, whereas the exchange energy is not: it contains the term $1/r_{s}$ even within the simplified DW model. This is in disagreement with the statement by Bernand-Mantel et al. [21] that the exchange energy is linearly proportional to $r_{s}$. Note that the critical value of $d_{c}=2/\pi$, two times smaller than $d_{c}=4/\pi \approx 1.273$, was calculated for the isolated chiral skyrmions in infinite films in zero external magnetic field by Kiselev et al. [12], and later this value was corrected by Leonov et al. [13] to be $d_{c}=1.224$.

In the limit of small DMI strength *d* << 1, $r_{s}\left(d\right)$ cannot be directly determined from the equation $r_{s}\left(d\right)=dF\left(0\right)/2F_{a}\left(0\right)$ because F(0)=4 is finite, but $F_{a}\left(x\to 0\right)\to \infty$ is singular. The non-analytic behavior of the function $F_{a}\left(x\right)$ at x→0 can be approximately presented as $F_{a}\left(x\right)=F_{a}\left(1\right)/x^{\alpha }$. To calculate ξ(d), we need to analyze the exchange energy. The approximate Equation (3b) has very good accuracy at ξ≥2, but it predicts a wrong asymptotic behavior at ξ→1+0 and the exact Equation (3a) should be used instead within this limit. We rewrite the exchange energy in the form

(7) $$E_{ex}\left(x\right)=x^{2}{ℑ}_{1}\left(x\right)+2x{ℑ}_{0}\left(x\right)+2{ℑ}_{-1}\left(x\right),\;{ℑ}_{n}\left(x\right)=\int_{0}^{\infty }d\rho \rho^{n}\frac{1}{\cosh^{2}f\left(\rho ,x\right)}.$$

The functions ${ℑ}_{n}\left(x\right)$ are not analytic at x=ξ−1→0, but it is possible to calculate ${ℑ}_{0}\left(0\right)=\pi$ and ${ℑ}_{-1}\left(x\right)={ℑ}_{-1}\left(0\right)+{{ℑ}^{\prime }}_{-1}\left(0\right)x$, ${ℑ}_{-1}\left(0\right)=2$, ${{ℑ}^{\prime }}_{-1}\left(0\right)=-\pi$. Therefore, the asymptotic behavior of the function $E_{ex}\left(x\right)$ is determined by the first term in Equation (7), $E_{ex}\left(x\right)=4+x^{2}F_{a}\left(x\right)=4+F_{a}\left(1\right)x^{2-\alpha }$, $F_{a}\left(x\right)\equiv {ℑ}_{1}\left(x\right)$. Using this expression, we can solve Equation (6) in the limit x=ξ−1→0 and get $\xi \left(d\right)=1+{\left(d/\kappa^{1/2}\right)}^{1/\left(1-\alpha \right)}$, where $\kappa =4\left(2/\alpha -1\right)F_{a}^{2}\left(1\right)/F^{2}\left(0\right)$. Then, from the equation $r_{s}\left(d\right)=dF\left(x\right)/2F_{a}\left(x\right)$, accounting F(0)=4, we immediately get the expression $r_{s}\left(d\right)=\Delta \left(d\right)=2F_{a}^{-1}\left(1\right)\kappa^{\alpha /2\left(\alpha -1\right)}d^{1/\left(1-\alpha \right)}$. Numerical calculation of the asymptote of $F_{a}\left(x\to 0\right)$ showed that the exponent α=2/3, and $F_{a}\left(1\right)=1.121$. Therefore, the skyrmion radius calculated within the generalized DeBonte model at d<<1, $r_{s}\left(d\right)=\Delta \left(d\right)=\left(4/F_{a}^{3}\left(1\right)\right)d^{3}$, is essentially smaller than the radius predicted by the generalized DW model. The skyrmion energy is $E\left(d\right)=4\left[1-F_{a}^{-3}\left(1\right)d^{4}\right]$. It is slightly higher than the DW model energy $E_{DW}\left(d\right)=4\left[1-{\left(d/d_{c}\right)}^{2}/2\right]$. This is not a surprise because the DW model containing integration in the vicinity of $r_{s}$ always underestimates the skyrmion energy at $d<d_{c}$.

**Figure 1:**
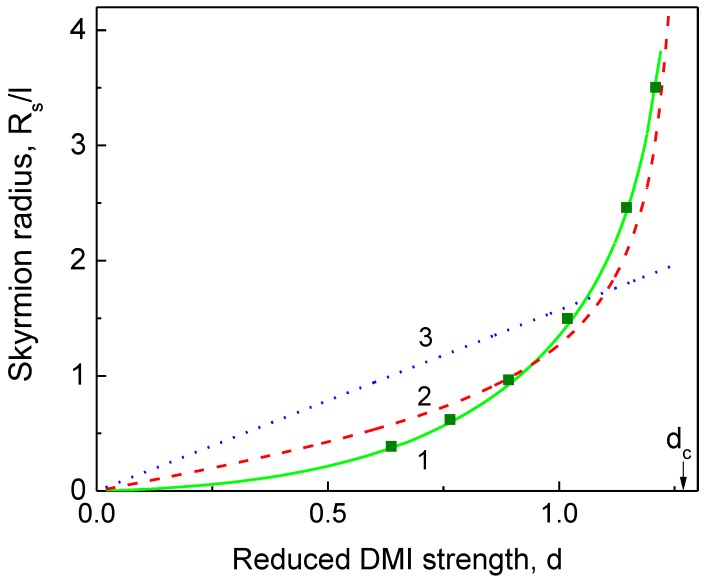
The skyrmion radius in units of $l=\sqrt{A/K}$ vs. the Dzyaloshinskii–Moriya exchange interaction (DMI) strength, d=|D|l/A: (1) Generalized DeBonte ansatz (solid green line); (2) generalized domain wall (DW) ansatz [15] (dashed red line); (3) linear ansatz [12] (dotted blue line). The radius obtained from numerical minimization of the skyrmion energy (2) is shown by deep green squares.

**Figure 2:**
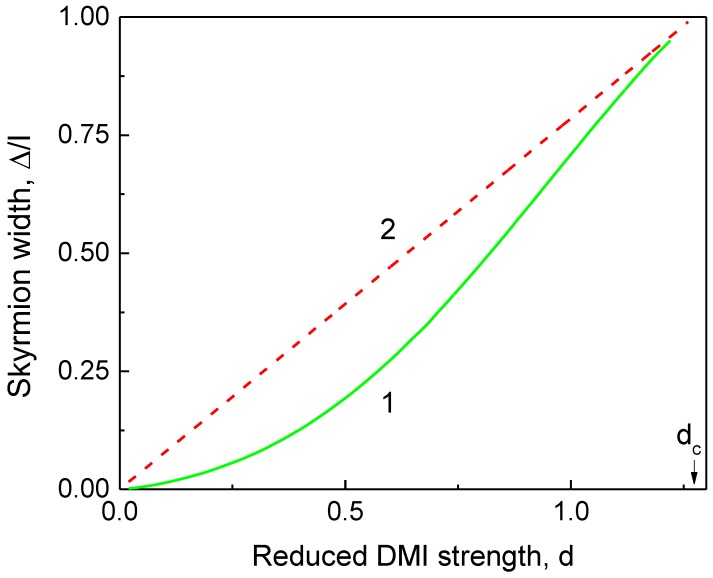
The skyrmion width in units of $l=\sqrt{A/K}$ vs. the DMI strength, d=|D|l/A: (1) Generalized DeBonte ansatz (solid green line); (2) generalized DW ansatz [15] (dashed red line).

**Figure 3:**
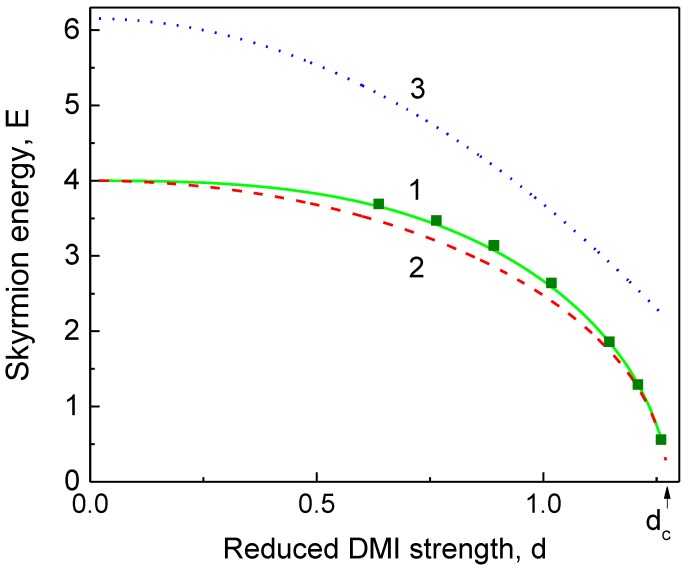
The skyrmion energy in units of 2πAL vs. the DMI strength, d=|D|l/A: (1) Generalized DeBonte ansatz (solid green line); (2) generalized DW ansatz [15] (dashed red line); (3) linear ansatz [12] (dotted blue line). The numerical minimization of the skyrmion energy (2) is shown by deep green squares.

The equilibrium skyrmion radius, width, and energy vs. the DMI strength are shown in Figure 1, Figure 2 and Figure 3, respectively.

The DW ansatz and linear skyrmion ansatz result in the incorrect dependence $r_{s}\left(d\right)$, especially at small *d* (d<1) (see Figure 1). The generalized DW model [15] predicts the skyrmion width for intermediate values of *d*, which is approximately two times larger than one calculated within the generalized DeBonte ansatz (see Figure 2). The skyrmion energies calculated within the DeBonte and DW models are very close for $0<d<d_{c}$, whereas the linear model [12] overestimates the skyrmion energy up to 50% and predicts the wrong value of $d_{c}$ (see Figure 3). The skyrmion radius $r_{s}\left(d\right)$ (Figure 1) and skyrmion energy (Figure 3) calculated analytically using the DeBonte ansatz and numerically practically coincide.

Above, we calculated the stability of the chiral Bloch and Neel skyrmion magnetization configurations in ultrathin films as a function of the DMI strength. The second derivative of the skyrmion energy (5) $\partial^{2}E/\partial r_{s}^{2}=2F_{a}\left(x\right)>0$. Therefore, the sufficient condition of existence of the skyrmion local energy minimum $\left(\partial^{2}E/\partial r_{s}^{2}\right)\left(\partial^{2}E/\partial \xi^{2}\right)-{\left(\partial^{2}E/\partial r_{s}\partial \xi \right)}^{2}>0$ is satisfied for the skyrmion solution ξ(d), $r_{s}\left(d\right)$ within the interval $0<d<d_{c}$. The isolated skyrmions are metastable within a range of the values of d satisfying the inequality $d<d_{c}$ and do not exist at $d>d_{c}$ (the skyrmion minimum transforms to an energy maximum at $d=d_{c}$).

To describe skyrmion magnetization analytically we used the DeBonte radial domain wall ansatz [19], the accuracy of which was numerically checked for circular dots in Ref. [22]. The calculated equilibrium skyrmion radius $R_{s}\left(d\right)$ and the skyrmion width Δ(d) increase with increasing DMI strength (Figure 1). However, the continual model becomes inaccurate for the sizes below 1 nm. The typical values of A= 10 pJ/m and K= 0.1 MJ/m^3^ yield the magnetic length l = 10 nm for ultrathin films. The conditions $R_{s}\left(d\right)\geq$ 1 nm, Δ(d)≥1 nm mean that the continual model can be applied if the reduced DMI strength d≥ 0.2 or |D|≥ 0.2 mJ/m^2^ in absolute units. Simulations [23] within a discrete model on a simple cubic lattice with period a showed that the skyrmion state collapses to the uniformly magnetized state at $R_{s}\approx \left(4÷5\right)a$ or $R_{s}\approx$1.0–1.3 nm for Co. We note that in restricted geometry (circular dots) the skyrmion radius dependence on the DMI strength $R_{s}\left(d\right)$ has an inflection point at $d\approx d_{c}$ [17,24] and the skyrmion width Δ(d) reveals a broad maximum in the vicinity of $d_{c}$ [24]. The typical value of DMI strength, *D*, accessible in experiments with ultrathin films like X/Co (X = Pt, Ir, Pd) is 1–2 mJ/m^2^ [8,9,10,11]. Therefore, all observed Neel skyrmions in these nanostructures are metastable (if $d<d_{c}$) or unstable (if $d>d_{c}$) in a zero out-of-plane magnetic field. To compare the calculated skyrmion sizes with the experimental data [8,9,11], we used the experimental values of the exchange stiffness, *A*, the effective magnetic anisotropy constant, *K*, the DMI strength, *D*, and the skyrmion radius, *R_s_*. Using the set *A* = 10 pJ/m, *K* = 0.17 MJ/m^3^, *D* = 1.6 mJ/m^2^ for Ir/Co/Pt multilayer films taken from Ref. [9], we calculated the parameters $l=\sqrt{A/K}$ = 7.7 nm, and d=|D|l/A = 1.227. The measured skyrmion radius in the smallest out-of-plane magnetic field of 12 mT was $R_{s}^{\exp}$ = 40 nm, or $r_{s}^{\exp}=R_{s}^{\exp}/l$ = 5.2. The calculations within the generalized DeBonte model yielded the value $r_{s}^{cal}$ = 4.0. The measured value in Ref. [8] of $K_{u}=$ 1.37 MJ/m^3^ for Pt/Co/MgO film allows calculation of the effective anisotropy constant $K=K_{u}-\mu_{0}M_{s}^{2}/2$ = 0.138 MJ/m^3^, assuming the Co-layer saturation magnetization $M_{s}$ = 1400 kA/m. The value of the DMI strength measured by a Brillouin light scattering [8] is *D* = 2.05 mJ/m^2^. Given that the Co layer is relatively thick (i.e., 1.0–1.1 nm), we used the Co exchange stiffness constant close to its bulk value, *A* = 20 pJ/m, and calculated l= 12.0 nm and d= 1.234. The measured skyrmion radius was $R_{s}^{\exp}$ = 65 nm, or $r_{s}^{\exp}=R_{s}^{\exp}/l$ = 5.4, whereas the calculations yielded the value $r_{s}^{cal}$ = 4.2. Using the experimentally found values of K= 0.24 MJ/m^3^, *D* = 2.0 mJ/m^2^, and the estimated value of *A* = 11 pJ/m for Co/Pd multilayer films [11], we could calculate l= 6.8 nm and d= 1.231. The skyrmion radius, measured by Lorentz transmission electron microscopy [11], is $R_{s}^{\exp}$ = 45 nm, or $r_{s}^{\exp}=R_{s}^{\exp}/l$ = 6.6, whereas the calculations yielded the value $r_{s}^{cal}$ = 4.1. The agreement of the skyrmion sizes measured by X-ray imaging [8,9] and by our calculations is reasonably good. The skyrmion size measured by Lorentz transmission microscopy was larger than the calculated one. This can be explained by the different mechanisms of image formation in these experiments. The image contrast is proportional to the magnetization out-of-plane component m⋅z for the X-ray imaging [8,9,10], whereas the contrast is proportional to the out-of-plane component of the magnetization curl (∇×m)⋅z, for Lorentz microscopy imaging. The parameters K and D can be extracted with reasonable accuracy from independent experiments. The exchange stiffness A is poorly defined for ultrathin films with ferromagnetic layer thickness 0.5–1 nm. The skyrmion sizes measured in Refs. [8,9,11] are quite large, 40–65 nm. This means that the DMI parameter d is also large and close to its critical value $d_{c}$, and the value of the skyrmion radius is very sensitive to the exact value of d (see Figure 1). According to its definition $d=\left|D\right|/\sqrt{AK}$, the DMI parameter depends on A. This leads to an uncertainty in the interpretation of the experimental data [8,9,11]. This uncertainty may lead to the case $d>d_{c}$ for Ir/Co/Pt multilayer films [9]. Decreasing the out-of-plane magnetic field can essentially increase the skyrmion sizes (see Figure 2 in ref [9]), indicating that the single skyrmion state is unstable in zero out-of-plane field. We note that the dependences of the skyrmion radius $R_{s}$ on the DMI strength D for different values of A, simulated in Ref. [11], can be reduced to the universal curve $R_{s}\left(d\right)$ presented in Figure 1 if one changes the variable D to dimensionless variable d.

The case of magnetic dots considered in Refs. [14,17,22,24,25] is more complicated because the skyrmion configuration can be the dot ground state. The Neel skyrmions in circular dots can be metastable or stable even at $D>D_{c}=\left(4/\pi \right)\sqrt{AK}$. The calculated value of D for a transition between the metastable and stable Neel skyrmions in ultrathin circular dots is 1.5–2 times larger than one for infinite films for weak effective magnetic anisotropy $2K/\mu_{0}M_{s}^{2}<<1$ [14]. It was also shown that the Bloch skyrmions can be the dot ground state for in-plane magnetic anisotropy K<0 and D=0 [25].

In the investigated case of out-of-plane effective magnetic anisotropy *K* > 0, the large values of the Dzyaloshinskii–Moriya interaction strength $D>D_{c}$ cause the nucleation of more complicated magnetization configurations (nπ-skyrmions [17], spin spirals, labyrinth domain, etc.), that is, the individual Neel or Bloch magnetic skyrmion state with the topological charge |N|≈1 is no longer metastable.

## 4. Conclusions

We found that the isolated Bloch and Neel skyrmions in ultrathin magnetic films are metastable within the range of the DMI strength $0\leq d<d_{c}$, where $d_{c}=4/\pi$ or $D_{c}=4A/\pi l$ in absolute units, A is the material exchange stiffness, and $l=\sqrt{A/\left(K_{u}-\mu_{0}M_{s}^{2}/2\right)}$ is the material magnetic length. The calculated skyrmion radius $R_{s}$ increases as d increases and diverges at $d\to d_{c}-0$, whereas the skyrmion width Δ increases monotonically as d increases without any singularities at $d\to d_{c}-0$. The calculated skyrmion width is essentially smaller than the one calculated within the generalized domain wall model. The generalized DeBonte ansatz is a very good approximation to calculate the skyrmion radius, width, and energy. The linear skyrmion model cannot be applied for quantitative analysis of the skyrmion energy and size.
